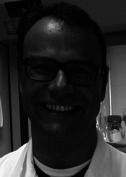# Activity‐Based Protein Profiling (ABPP) of Oxidoreductases

**DOI:** 10.1002/cbic.202000542

**Published:** 2020-10-20

**Authors:** Rita Fuerst, Rolf Breinbauer

**Affiliations:** ^1^ Institute of Organic Chemistry Graz University of Technology Stremayrgasse 9 8010 Graz Austria; ^2^ BIOTECHMED Graz Austria

**Keywords:** chemical proteomics, covalent inhibitors, drug discovery, oxidoreductases, proteomics

## Abstract

Over the last two decades, activity‐based protein profiling (ABPP) has been established as a tremendously useful proteomic tool for measuring the activity of proteins in their cellular context, annotating the function of uncharacterized proteins, and investigating the target profile of small‐molecule inhibitors. Unlike hydrolases and other enzyme classes, which exhibit a characteristic nucleophilic residue, oxidoreductases have received much less attention in ABPP. In this minireview, the state of the art of ABPP of oxidoreductases is described and the scope and limitations of the existing approaches are discussed. It is noted that several ABPP probes have been described for various oxidases, but none so far for a reductase, which gives rise to opportunities for future research.

## Graphical abstract

**Off the beaten track**: Activity‐based protein profiling (ABPP) is a technique that enables functionally active enzymes to be studied. The specific molecular design of the probe allows the study of different families of enzymes. This minireview describes the successes and challenges of using ABPP to study oxidoreductase enzymes, which still represent an underexplored goal in ABPP.

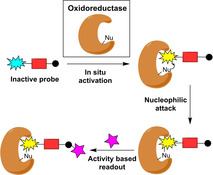

## 1 Introduction

Since the completion of the Human Genome Project, the time and costs for the sequencing of genes and even genomes have fallen at such a fast pace that it has been compared to the continuous development of microchip capacity following Moore's law.^[1]^ Every single day the databases for deposited gene sequences are filled with new entries. Furthermore, today it is even possible to quantify the gene expression of individual cells via RNAseq analyses.^[2]^ Similarly, mass‐spectrometry based proteomics have seen an overwhelming rise, which has enabled proteomic analysis to be performed on a single‐cell level.[^3^, ^4^] However, neither gene sequence information nor quantification of protein abundance is sufficient to describe the status of the active proteome of a cell or living organism. In order to address this limitation, activity‐based protein profiling (ABPP) has been developed. ABPP aims to only measure proteins, which are able to exert a catalytic function. Pioneered by Cravatt,^[5]^ Bogyo,^[6]^ and others, this approach has become a very valuable tool in chemical proteomics over the past two decades,[^7^, ^8^, ^9^, ^10^, ^11^] allowing the investigation of the mode of action and selectivity profile of drugs[^12^, ^13^, ^14^, ^15^] and natural products.[^16^, ^17^] It has been used to determine the activity profile of proteins in fields ranging from microbiology[^18^, ^19^] to plant sciences.^[20]^ Importantly, ABPP enables the functional annotation of uncharacterized proteins. This complements ‐ and sometimes corrects ‐ the putative annotation of proteins via sequence comparison, in which historical functional assignment gets perpetuated but not validated.

At the heart of any ABPP effort is the design of an activity‐based probe. It includes a warhead,^[21]^ which ‐ if not already reactive enough ‐ will be activated by the protein class it has been designed for and transformed into a highly reactive (typically electrophilic) species. In a subsequent step, the warhead will be attacked by nucleophilic residues of the protein of interest. The covalently modified protein can then be visualized and analyzed by various types of labels. ABPP has shown its value for certain enzyme families, such as serine hydrolases, threonine hydrolases, cysteine hydrolases,^[22]^ or glycosidases.^[23]^ These enzymes are distinguished by a characteristic nucleophilic group responsible for their catalytic function. It remains a goal of ABPP to extend this approach to other enzyme families. A particularly attractive goal would be the ABPP of oxidoreductases.

The enzyme class of oxidoreductases (EC 1) comprises many functionally different enzymes. Some members are responsible for essential metabolic processes, and others are involved in the synthesis and processing of secondary metabolites. A more detailed insight into the function of oxidoreductases would not only stimulate the understanding of the biochemistry of cells but would also allow to correlate the activity of these enzymes with cellular disease states and offer opportunities for utilizing them in biocatalysis and biotechnology. More than other enzyme classes, oxidoreductases are relying on cofactors such as flavins, NADH, NADPH, PLP, heme, etc. in pursuing its catalytic function. As a consequence, it is more difficult ‐ and sometimes impossible ‐ to annotate their function on a gene sequence level as the encoded amino acids are not characteristic for their catalytic function. Therefore, a chemical proteomics platform such as ABPP would be of tremendous value to fill this gap to monitor individual oxidoreductases and to discover new ones. This article aims to review the current status of this field.

## 2 Activity‐Based Protein Profiling of Various Oxidoreductases

So far only a rather limited set of ABPP probes has been developed to investigate oxidoreductases. In the following sections the probes will be discussed in the context of the enzyme family, which they were designed to address.

### 2.1 Cytochrome P450

Cytochrome P450 enzymes catalyze a variety of chemical transformations, such as C‐hydroxylations, epoxidations, heteroatom oxygenations, dealkylations, etc. These membrane‐bound monooxygenases play an important role in the metabolism of drugs, xenobiotics and endogenous signaling compounds (e. g. eicosanoids). There are 18 mammalian cytochrome P450 (CYP) families, which encode 57 genes in the human genome.^[24]^ The sequence homology within these genes is as low as 16 %. Many of these oxidases are strongly regulated by substrate binding or post‐translational modifications. Therefore, neither gene expression nor protein abundance reflects the active state of these oxidases. In 2007 Wright and Cravatt developed an ABPP approach to characterize several of these proteins.^[25]^ Starting from the known broad‐spectrum mechanism‐based P450 inhibitor 2‐ethynylnaphthalene, activity‐based probe **1** was developed by introducing a versatile alkyne handle, which allows selective tagging, detection, enrichment and identification of the protein–probe conjugate by Cu‐catalyzed azide‐alkyne coupling.[^26^, ^27^]

**Scheme 1:**
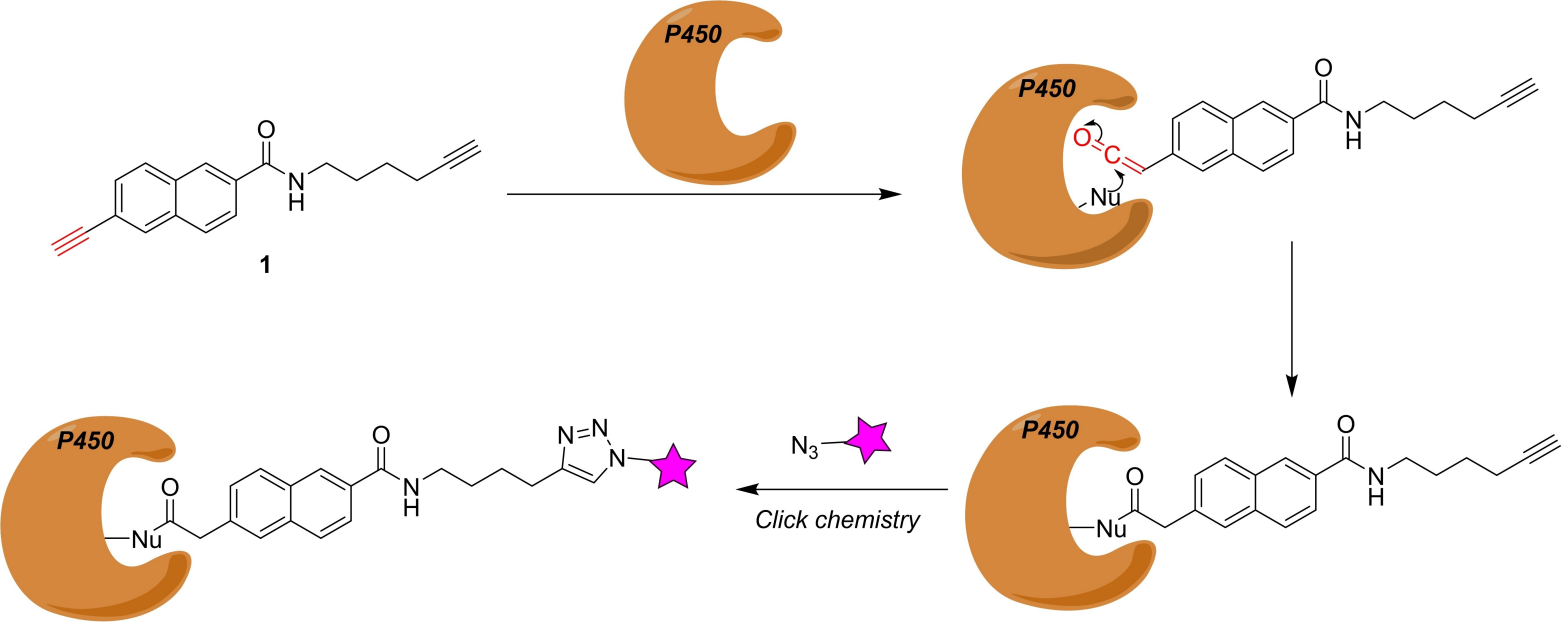
Design of the mechanism‐based P450‐probe **1**. The warhead is depicted in red.

The alkyne warhead^[28]^ is first converted into a highly electrophilic ketene by a P450 oxidase. Next, the ketene gets attacked by nucleophilic residues of the P450 enzyme leading to a covalent protein‐probe adduct, which can be labeled with a fluorescent dye using Cu^I^‐catalyzed azide‐alkyne coupling (Scheme 1). It could be demonstrated that compound **1** selectively labels P450 oxidases in mouse liver microsomes. In particular, probe **1** showed activity against P450 1a2, 3a11, 2c29 and 2d9/2d10. The probe proved also to be useful for in vivo labeling of P450 enzymes in mice. Through development of a variant of **1** that possesses a linker group terminating in an azide, the authors gained the evidence that the aryl‐alkyne functional unit is likely the primary reactive moiety for inactivating P450s.

**Figure 1:**
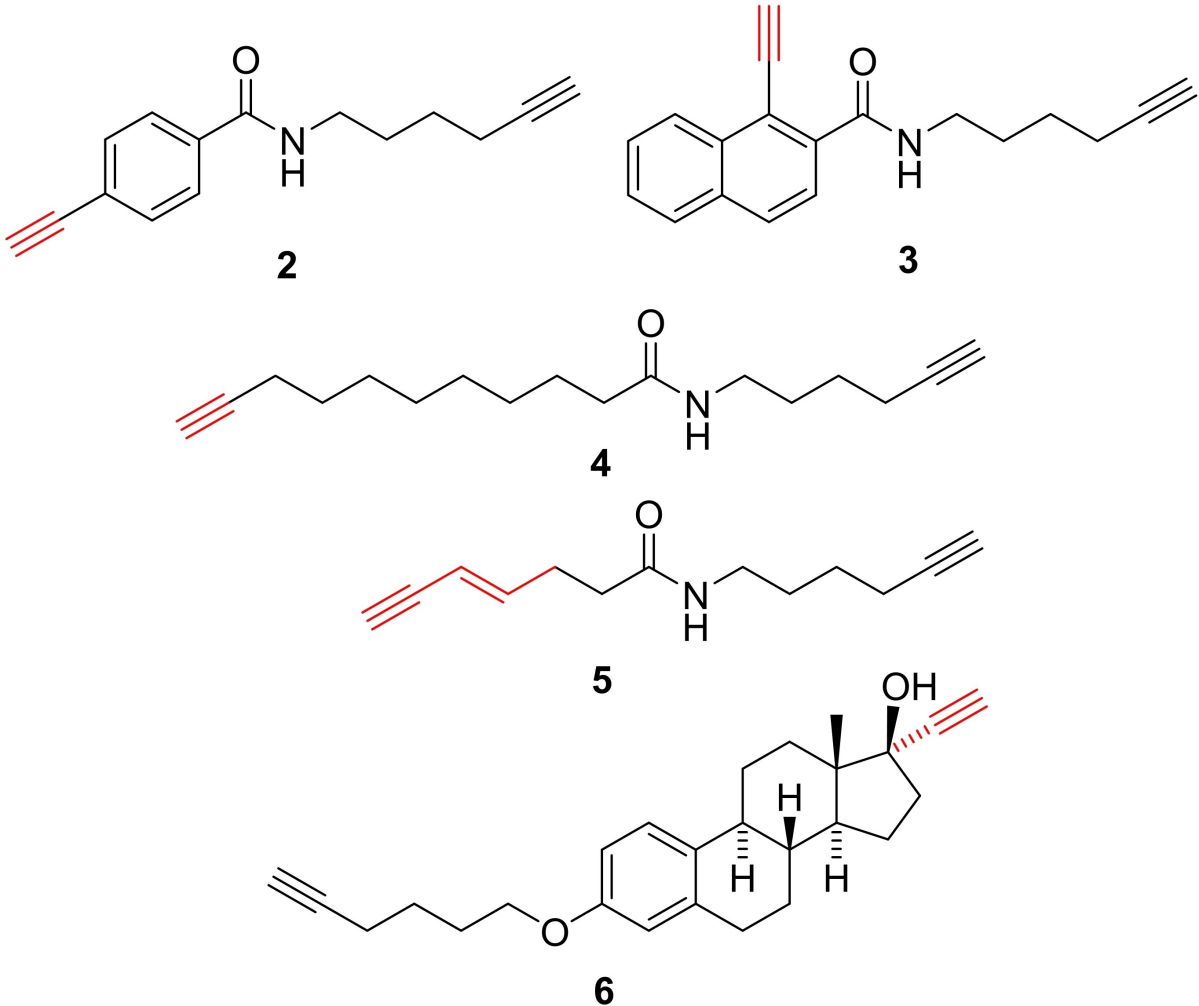
Selected probes designed for the ABPP of human P450s. The warheads are depicted in red.

As P450 monooxygenases catalyze various oxidation reactions, the Cravatt group presented in a subsequent publication a panel of ABPP probes to study these functions.^[29]^ Among others, the probes **2** and **3** represented arylalkynes (which will be activated to ketenes similar to probe **1**), while the aliphatic alkynes **4** and **5** would allow for a different type of P450 reactivity in which twofold hydroxylation in the propargylic and allylic position, respectively, would give rise to alkynylketones, which would provide strong Michael acceptor electrophiles. The design of probe **6** was inspired by the fact that the oral contraceptive 17‐α‐ethynylestradiol is known to inactivate several human P450s (Figure 1). The probes were tested against several human P450 enzymes. While probes **1**–**4** showed quite promiscuous behavior, probes **5** and **6** showed labeling of individual P450s. With the ABPP approach the authors could also demonstrate that the addition of aromatase inhibitors, formestane and anastrozolole, inhibited but in one case also increased labeling of certain P450 oxygenases, indicating unanticipated drug–drug interactions.

Wright used the panel of P450 ABPP probes **1**–**6** to investigate the effect of high‐fat diet‐induced obesity on the P450 activities in the liver and lungs of mice.^[30]^ Their data showed significantly reduced activities of hepatic P450s in obese mice, which also led to a decreased capacity for bile acid synthesis.

In a different study, they used probes **1**, **2** and **4** together with ABPP probes against ATPases and proteases to characterize protein activities within the developing murine lung and could observe increased activity of many P450s primarily in the postnatal lung.^[31]^

**Figure 2:**
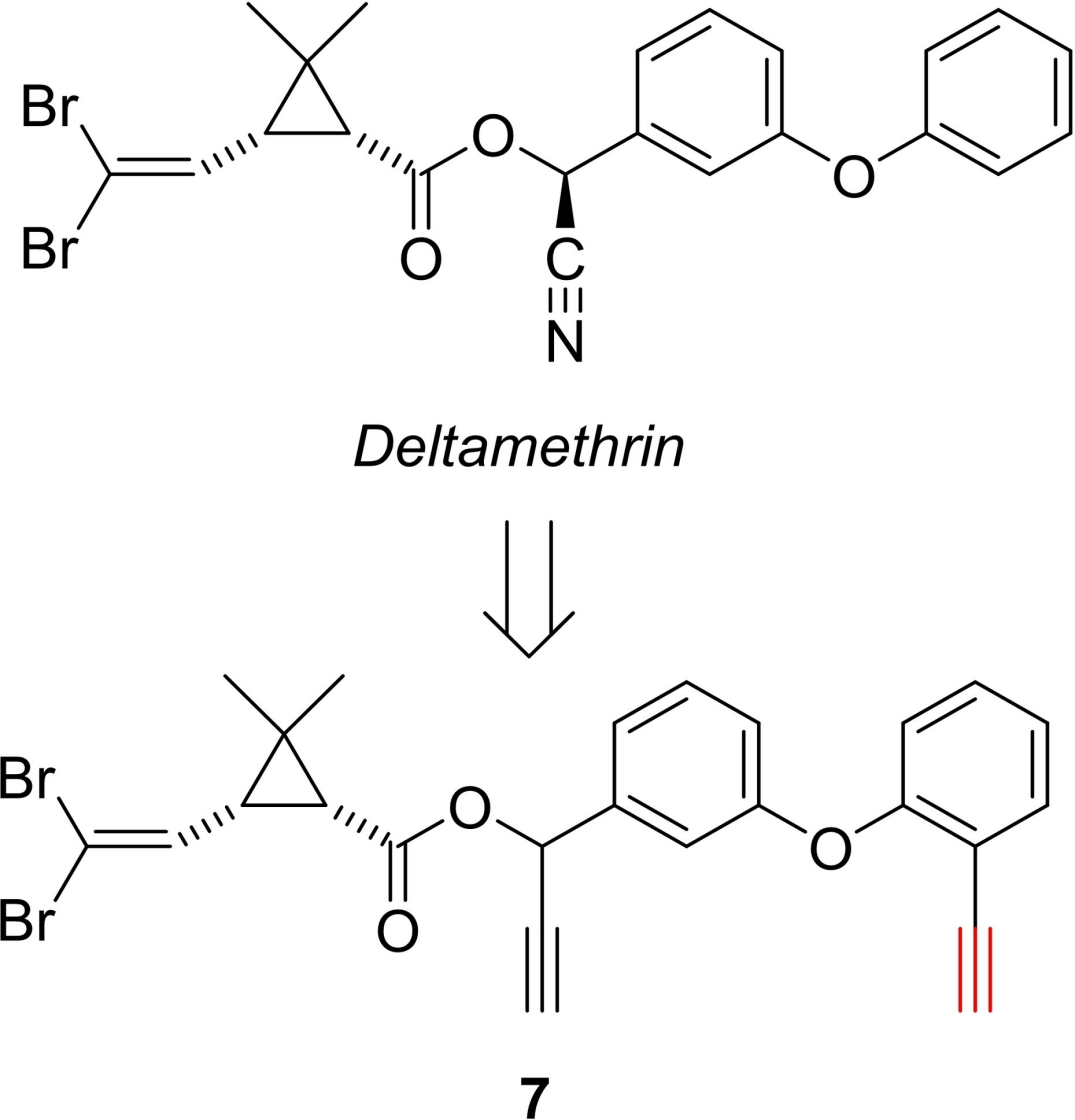
Probe **7** developed for profiling cytochrome P450 activities associated with pyrethroid insecticide interactions. The warhead is depicted in red.

Paine's group of has prepared ABPP probes derived from the pyrethroid Deltamethrin to investigate the metabolism of these widely used insecticide class in insects. The ABPP approach should give insight into the increasingly observed resistance in malaria‐transmitting mosquitos. Upon several probes, compound **7** proved as especially useful. The basis for the design of compound **7** was Deltamethrin. The cyano group was replaced by an alkyne click handle and an alkyne warhead was placed in ortho‐position of the phenyl ether group (Figure 2).^[32]^ Compound **7** selectively labeled the recombinant mosquito P450 CAP6M2. The probe was further used to determine the interactome (“pyrethrome”) in rat liver, identifying pyrethroid‐metabolizing enzymes.

**Figure 3:**
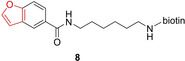
Benzofuran‐derived Probe **8** for P450 labeling. The warhead is depicted in red.

Sellars et al. took inspiration of the known CYP inhibitor furanocoumarin to design the benzofuran‐derived probe **8**. A P450 enzyme could convert the substrate into an

epoxyfuran, which would be electrophilic enough to be attacked by protein nucleophiles (Figure 3).^[33]^ Probe **8** exhibited the most effective NADPH‐dependent binding to CYP3A4, which is also the most abundant P450 in the human body.

### 2.2 Monoamine oxidases

**Scheme 2:**
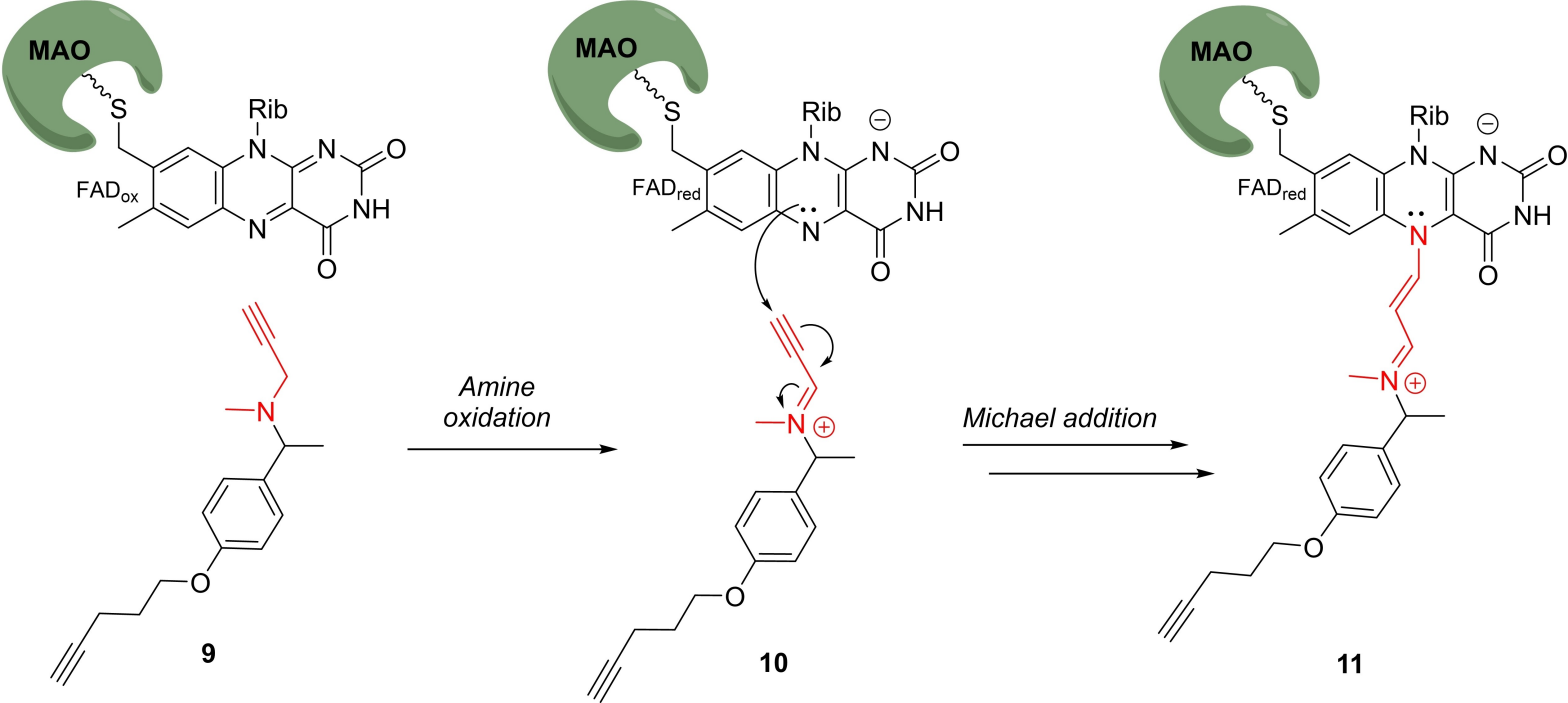
Design of the mechanism‐based MAO‐probe **9**. The warhead is depicted in red.

Flavin‐dependent enzymes catalyze a diverse set of chemical reactions encompassing oxidations, monooxygenations, dehydrogenations, reductions and halogenations. The intrinsic structural and functional diversity and the lack of conserved residues in the active site make them elusive to functional annotation by established genomic and proteomic analyses. In 2012, Breinbauer and Sieber presented the first ABPP‐approach of a flavin‐dependent oxidase by studying the activity of monoamine oxidases (MAO, EC 1.4.3.4).^[34]^ MAO are flavin adenine dinucleotide (FAD)‐containing enzymes, localized in the outer membrane of mitochondria, and catalyze the oxidative deamination of important neurotransmitters, including serotonin, norepinephrine and dopamine.^[35]^ There are two isoforms in humans (MAO A and B), which are associated with the development of cardiovascular and neurodegenerative disorders. Several MAO inhibitors are clinically used for the treatment of depression, Parkinson's disease, and other mental disorders. Breinbauer and Sieber converted the known MAO‐inhibitor and anti‐Parkinson drug deprenyl to the MAO‐ABPP probe **9**, by attaching a click handle at the phenyl ring *para* to the propargylamine warhead.[^34^, ^36^] Amine oxidation of **9** by MAO generates the strong iminium Michael acceptor **10**, which gets attacked nucleophilically by the reduced FAD cofactor (Scheme 2). As the FAD is covalently attached to MAO via an 8α‐(*S*‐cysteinyl) linkage a stable protein‐probe adduct (**11**) is formed, which could be visualized by click tagging. With this approach the authors could show that deprenyl reacts in human brain cancer cells quite selectively with MAO A and B. This outstanding selectivity is a consequence of the unique activation mechanism triggered by the idiosyncratic reaction mechanism of this enzyme class, and stands in contrast to many other CNS drugs, exhibiting a polypharmacological profile.

**Figure 4:**
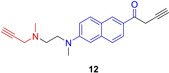
MAO B‐selective dual purpose probe **12**. The warhead for covalent attachment is depicted in red, the chromophore for live‐cell imaging in blue.

Activity‐based probes can also be used for the cellular imaging of MAOs.^[37]^ The group of Yao designed the dual‐purpose activity‐based probe **12**, which showed a formidable selectivity for MAO B (Figure 4).^[38]^ Compound **12** was used for the ABPP of MAO B and live cell bioimaging of MAO B activity in cell and tissue models of Parkinson's disease without encountering diffusion problems.

### 2.3 Flavin monooxygenases

**Scheme 3:**
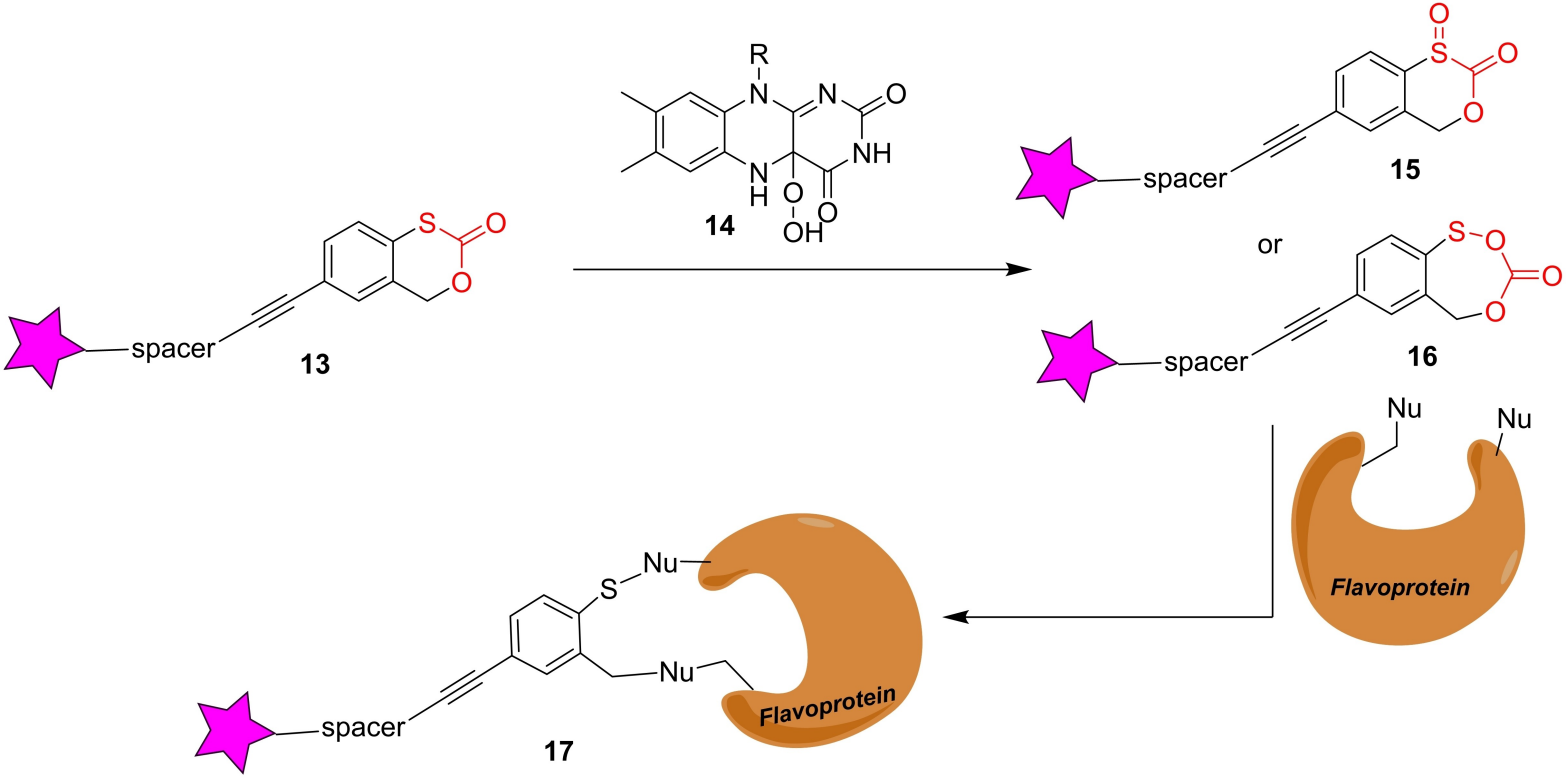
Labeling of flavin‐dependent oxidases with general probe **13**. The warhead is depicted in red.

Aerobic flavoenzymes are subcategorized into three major classes: oxidases, monooxygenase and halogenases. The group of Burkart has sought to develop a more universal probe for aerobic flavoenzymes and designed probe **13**, which features a cyclic thiocarbonate as a latent warhead (Scheme 3).^[39]^ In contrast to the MAO probes discussed above, **13** does not require the flavin to be covalently attached to the protein of interest. It is characteristic for the catalytic activity of flavoproteins that upon oxidation of reduced flavin the hydroperoxy‐containing flavin **14** is formed, which in the presence of **13** would produce the electrophilic acyl sulfoxide **15** or sulfenic anhydride **16**. These highly electrophilic intermediates would react with nucleophilic residues in the active site of the corresponding flavin‐dependent enzyme leading to covalent adduct **17**. It could be demonstrated that compound **13** was able to label the NRPS oxidase BpsA, a Baeyer‐Villiger cyclohexanone monooxygenase (CHMO) as well as the NRPS halogenase PltA, in in vitro experiments. PltA could also be labeled in an *E. coli* strain engineered to heterologously expressing PltA. Studies applying compound **13** to full proteomes or using the probe for the detection of flavoproteins at natural abundance have not been published.

### 2.4 Ammonia monooxygenase

**Figure 5:**
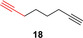
AMO‐probe **18**. The warhead for covalent attachment is depicted in red.

Ammonia monooxygenases (AMO) are a class of enzyme used by autotrophic bacteria^[40]^ and archaea^[41]^ to oxidize ammonia to hydroxylamine, which is further oxidized to nitrate by hydroxylamine dehydrogenase (HAO) completing the energy delivering nitrification process. AMOs are Cu‐dependent multimeric transmembrane enzymes, which are difficult to purify and whose structure has not yet been determined. Considerable insights into the activities and structure of AMOs have been gained from whole cell studies using different classes of inhibitors. The group of Hyman has introduced octa‐1,7‐diyne (**18**) as an ABPP probe to analyze AMO in the nitrifying bacteria *Nitrosomonas europea* (Figure 5).^[40]^ It was known that short chain alkynes inhibit AMO in a covalent manner. The second alkyne group was used as a click handle and allowed the fluorescent labeling of the protein on the SDS‐PAGE gel and its affinity enrichment via a biotin‐labeled enzyme–inactivator adduct. They could confirm that probe **18** labeled AmoA as its main target. Although the mechanism of inactivation was not outlined in the publication, previous evidence suggest that the alkyne gets oxidized to a ketene, which reacts with a nucleophilic residue of the protein.^[42]^

### 2.5 Myeloperoxidase

**Figure 6:**
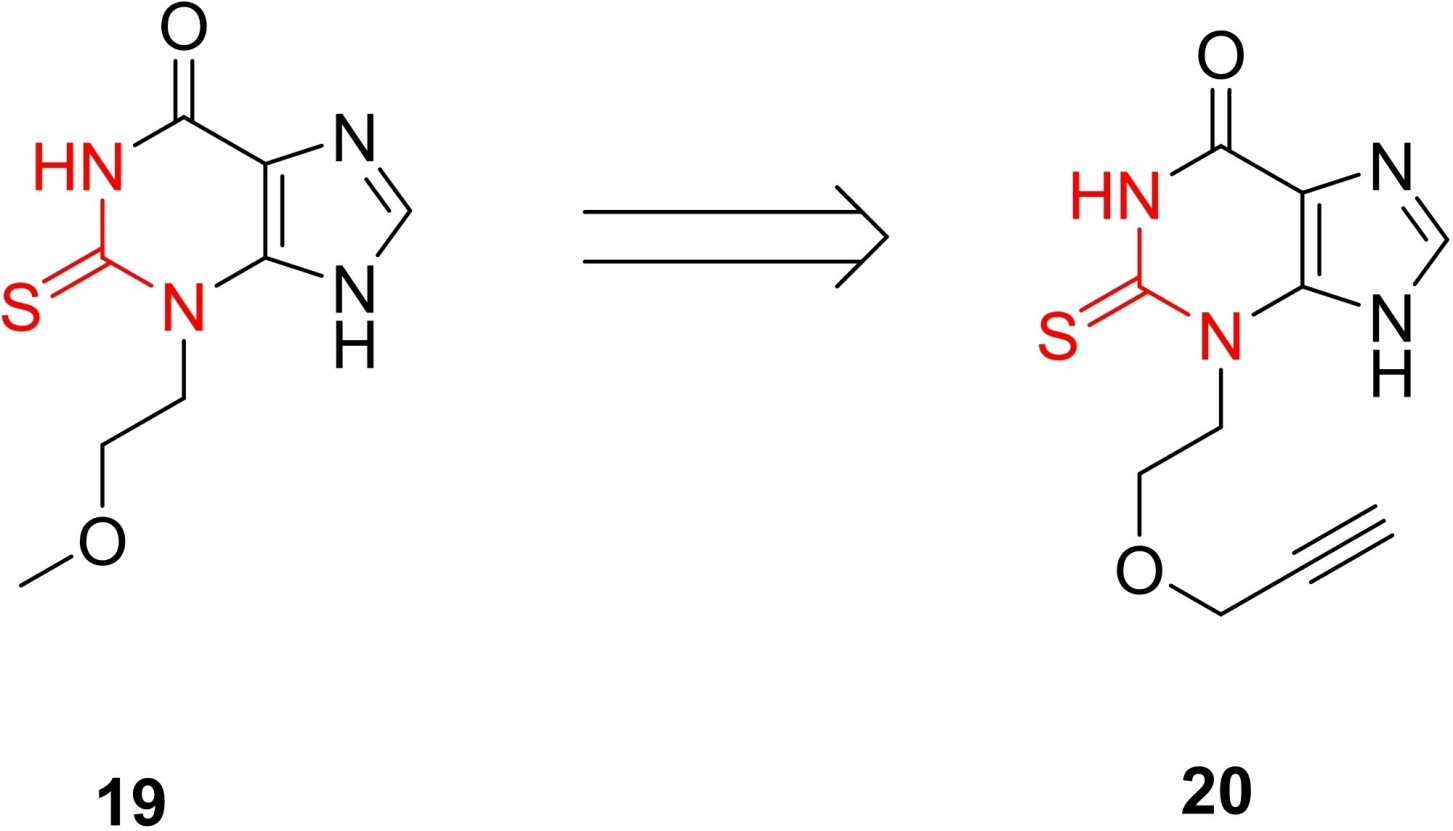
MPO‐probe **20**. The warhead for covalent attachment is depicted in red.

Myeloperoxidase (MPO, EC 1.11.1.7) is a heme peroxidase in neutrophils that catalyzes the synthesis of hypochlorous acid (HOCl) from chloride anions and H_2_O_2_. Whereas regular MPO activity is important for the normal host defense mechanism against pathogens, the persistent activation of MPO has been implicated in several disorders such atherosclerosis, rheumatoid arthritis, chronic obstructive pulmonary disease or neuroinflammation, making it attractive as a pharmacological target. Kettle and coworkers have identified 2‐thioxanthine (**19**) as a mechanism based inactivator of MPO.^[43]^ Experimental evidence strongly suggests that the Fe=O unit of activated MPO converts the thiourea unit of **19** into a radical. This reactive species ultimately induces a covalent linkage of the thioxanthine ring to the methyl group of the heme via a thioether bond. Ahn from Pfizer converted compound **19** into the ABPP probe **20** (Figure 6). This compound allowed them to study the protein profile of a structurally similar drug candidate.^[44]^ Compound **20** alone was unreactive towards soluble human and mouse liver proteomes, but labeled several protein targets from the same liver proteomes in the presence of MPO and H_2_O_2_. This result suggests that under artificially high MPO levels probe **20** is converted into an activated species, which is released from the enzyme and can attack other proteins.

### 2.6 Lipoxygenase

**Scheme 4:**
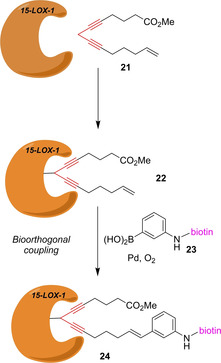
15‐LOX‐1 probe **21** and its biorthogonal labeling by oxidative Heck reaction. The warhead is depicted in red.

Lipoxygenases (LOX) are Fe‐containing dioxygenases, which catalyze the stereospecific insertion of molecular oxygen (O_2_) into polyunsaturated fatty acids, such as arachidonic acids. An important mammalian representative is 15‐lipoxygenase‐1 (15‐LOX‐1) playing a role in the biosynthesis of leuktorienes, lipoxins, 15‐HPETE, 15‐HETE and eoxins. 15‐LOX‐1 has gained attention as a drug target as its activity is associated with allergic airway diseases, atherosclerosis, cancer and various CNS diseases. The group of Dekker has designed the ABPP probe **21**, which mimics the natural polyunsaturated fatty acid substrate but contains a bispropargylic warhead (Scheme 4).^[45]^ A highly reactive allene radical is formed by single‐electron oxidation of the bis‐propargylic carbon atom, which leads to the covalent attachment of the probe to the active site of the enzyme forming adduct **22**. Instead of the commonly used azide–alkyne click chemistry, Dekker et al. used a biorthogonal oxidative Heck reaction^[46]^ with biotinylated phenylboronic acid **23** forming the biotinylated protein‐probe conjugate **24**. Using an alkene handle for bioconjugation facilitated the synthesis of the probe tremendously. Dekker et al. could demonstrate that compound **21** labels 15‐LOX‐1 in cell and tissue lysates.

### 2.7 Amine oxidases

**Scheme 5:**
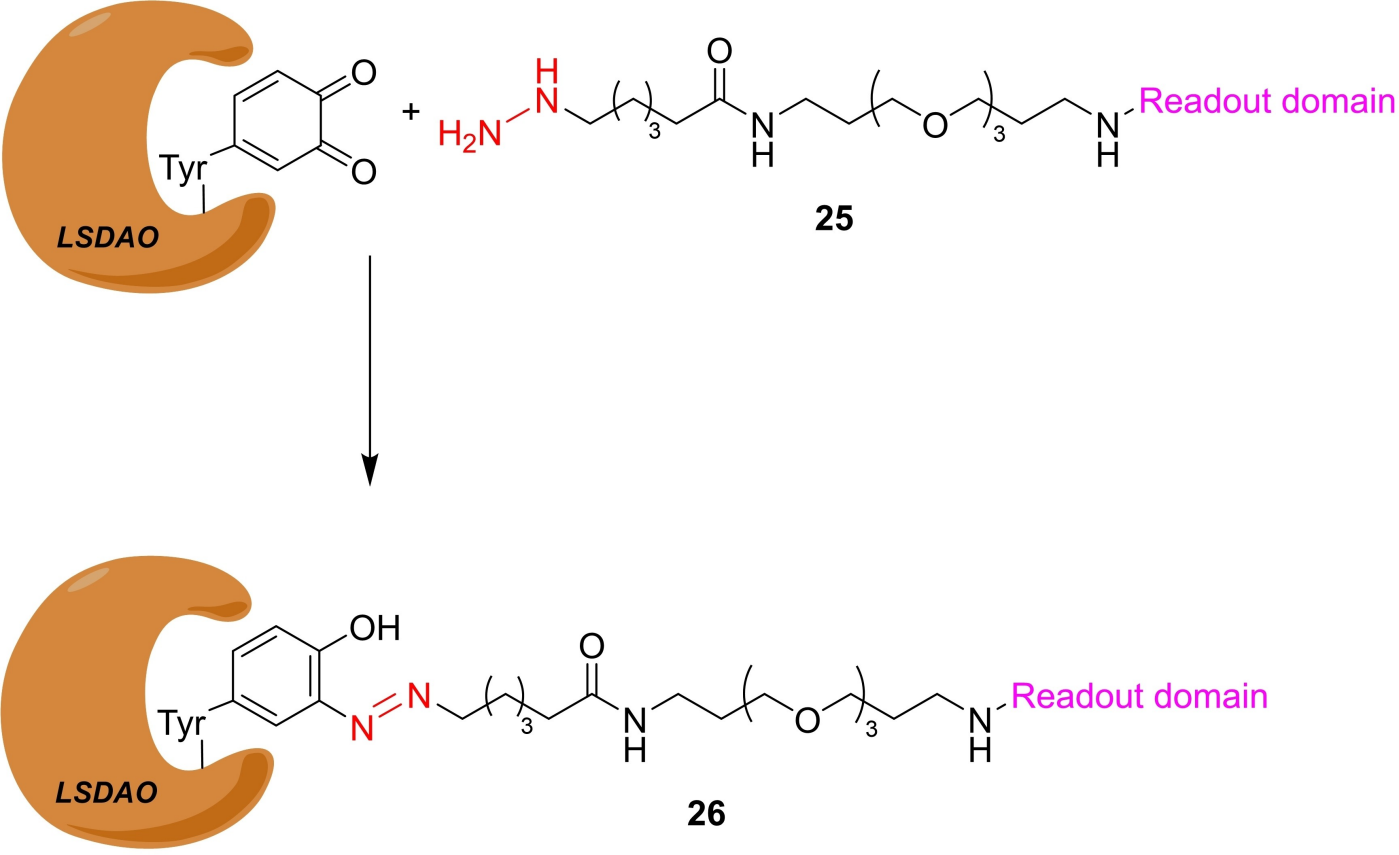
ABPP probe **25** for the visualization of LSDAO. The warhead is depicted in red.

Quinone‐dependent amine oxidases utilize a 1,2‐benzoquinone cofactor together with copper ions to oxidize aliphatic amines into aldehydes. The group of Jakobsche has developed ABPP probe **25** to visualize lentil seedling diamine oxidase enzyme (LSDAO; Scheme 5),^[47]^ which is described as a typical representative of the topoquinone subfamily. Fluorescein and 2,4‐dinitrophenyl were used as readout domains. It turned out, that the 2,4‐dinitrophenyl probe and subsequent western blotting produced the stronger signal with recombinant LSDAO. No experiments with cell lysates have been reported.

### 2.8 Aldehyde dehydrogenase

**Scheme 6:**
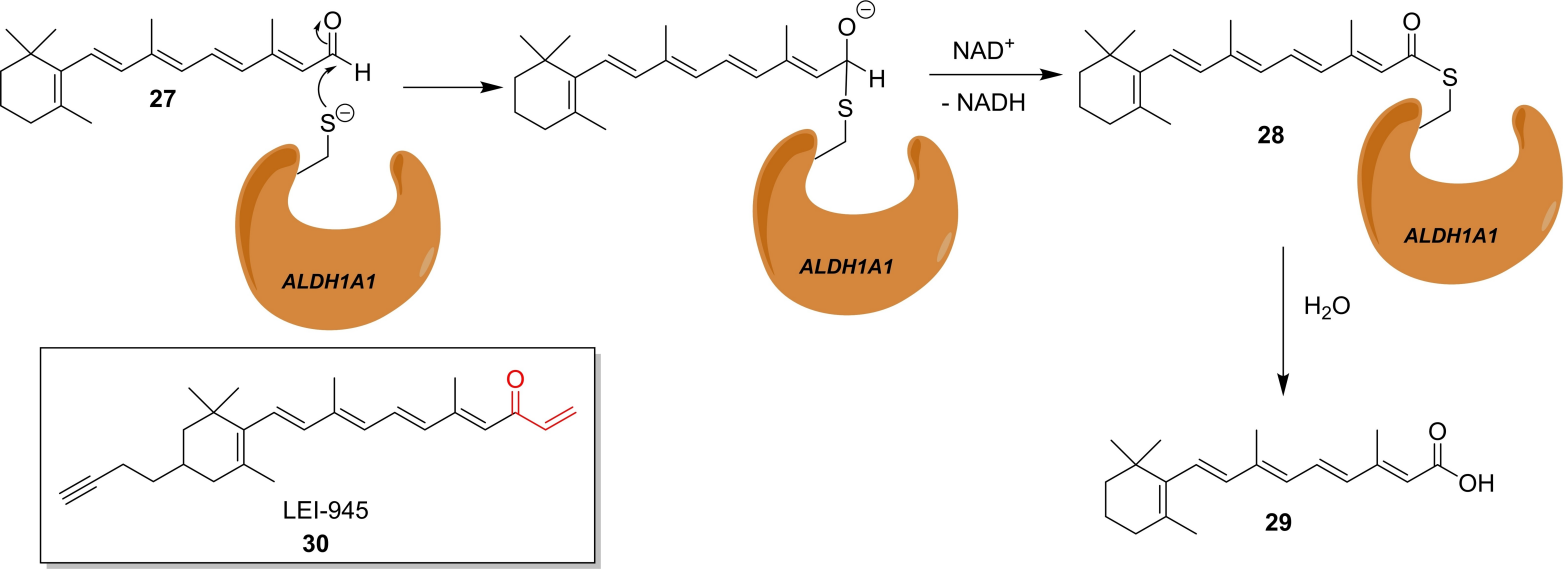
Oxidation of retinal to *all‐trans* retinoic acid (**27**) via ALDH1 A1 and the ABPP‐probe LEI‐945 (**30**). The warhead is depicted in red.

A crucial step in the biosynthesis of *all‐trans* retinoic acid (ATRA, **29**), the bioactive form of vitamin A, is the oxidation of retinaldehyde (**27**) catalyzed by three retinaldehyde dehydrogenases (ALDH1A1, ALDH1A2 and ALDH1A3). The oxidation involves the nucleophilic attack of a Cys of the dehydrogenase forming a thioacetal, which is oxidized to the thioester adduct **28**, followed by the release of compound **29** by hydrolysis (Scheme 6). ATRA (**29**) regulates several cellular functions by binding to the retinoic acid receptor modulating gene transcription. In particular, it influences cancer stem cell proliferation. By analyzing available structural information of ALDH1A1 the group of van der Stelt rationally designed ABPP‐probe LEI‐945 (**30**), which features a vinyl ketone warhead as a Michael acceptor addressing the reactive Cys residue and an attached alkyne handle for click chemistry tagging.^[48]^ It could be shown that compound **30** quite selectively binds to the three retinaldehyde dehydrogenases. Importantly, it does not react with other proteins known to have a “hyperreactive” cysteine,^[49]^ but show only cross reactivity with other proteins involved in retinal/retinoic acid biochemistry. Probe **30** enabled the comparative profiling of retinaldehyde dehydrogenase activity in a series of cancer cells.

**Figure 7:**
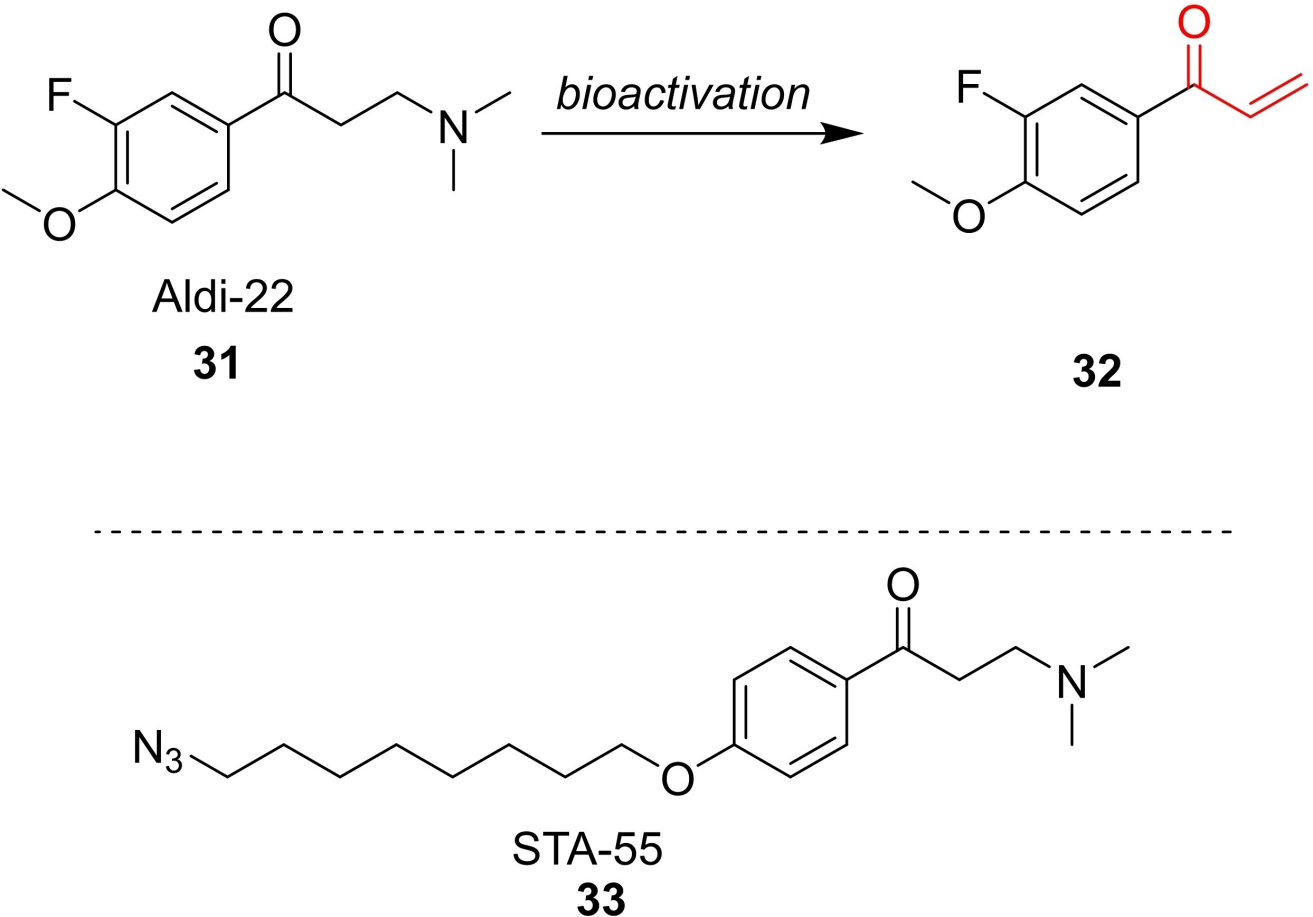
Broad‐spectrum aldehyde dehydrogenase ABPP probes **32** and STA‐55 (**33**) with a latent warhead.

Although LEI‐945 (**30**) proved to be useful as a probe specific for retinaldehyde dehydrogenases, it would also be desirable to have a broad‐spectrum probe for aldehyde dehydrogenases. The group of van der Stelt designed an ABPP probe based on the pan‐ALDH inhibitor Aldi‐2 (**31**), which has a masked warhead. Base‐induced elimination converts the β‐aminoketone into vinylketone **32**, an electrophilic trap for the reactive Cys in ALDHs. The corresponding pan‐ALDH ABPP probe STA‐55 (**33**) contains an azide click‐handle (Figure 7).^[50]^ STA‐55 (**33**) was used for the in situ selectivity profiling of three known ALDH‐inhibitors by competitive ABPP.

### 2.9 Miscellaneous approaches

More than 60 2‐oxoglutarate (2‐OG)‐dependent oxygenases are predicted in humans. These Fe‐containing enzymes play important roles in collagen biosynthesis, histone modification, lipid metabolism and hypoxic response.

**Figure 8:**
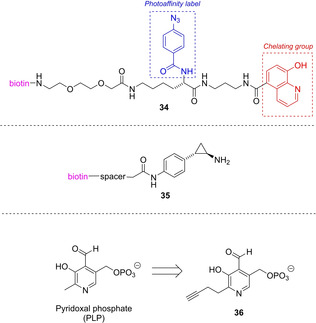
ABPP probes for 2‐OG oxygenases (**34**), LSD‐1 (**35**), and PLP‐dependent enzymes (**36**).

The group of Schofield has designed probe **34** (Figure 8),^[51]^ which exhibits a chelating group to achieve selective interaction with the Fe‐dependent enzymes and a photoaffinity label for covalent linkage. A biotin affinity tag allows the enrichment of the labeled proteins. The probe was used to profile 2‐OG oxygenases in a human cell line. Two histone lysyl demethylases (JARID1C, FBXL11) and the collagen lysyl hydroxylase LH3 emerged as clearly enriched proteins even at an endogenous level. The authors pointed out that their approach could be useful for identifying enzymes from the superfamily not already annotated as 2‐OG oxygenases. Future research should pay special attention in optimizing the chelating group in order to improve sensitivity and specificity of the probes.

Lysine‐specific demethylase‐1 (LSD‐1) is a FAD‐dependent amine oxidase acting on histones mediating transcriptional activation and repression. The group of Dekker designed ABPP‐probes for the detection of LSD‐1 activity based on the known inhibitor *trans*‐2‐phenylcyclopropyl‐1‐amine (tranylcypromine).^[52]^ In a series of control experiments they could show that their probe **35** was labeling LSD‐1, but when the samples were pre‐incubated with a LSD‐1 inhibitor they could not observe an activity dependence in the observed labeling (Figure 8).

Pyridoxal phosphate (PLP) is an enzyme cofactor used for various chemical transformations of biological amines in cellular processes, including the oxidation of amino acids to α‐keto acids. PLP‐dependent enzymes are evolutionary diverse, which makes its classification via sequence homology challenging. The group of Sieber has designed a pyridoxal analogue containing an alkyne click tag in the 2’‐position which is taken up by cells and phosphorylated to form the PLP analogue **36** (Figure 8),[^53^, ^54^] which was accepted by PLP‐dependent enzymes, and by reduction with NaBH_4_ led to the formation of stable conjugates. With this approach they could access 73 % of the current *S. aureus* PLPome and identified many more putative PLP‐dependent enzymes.^[53]^ Similarly, they could study the PLPome in human HEK293 cells and used it for target screening of Vitamin B_6_ antagonists.^[54]^

Bak et al. have developed a chemoproteomic platform to monitor selenocysteine reactivity by performing iodoacetamide‐labeling at low pH (pH 5.75), which allows them to suppress the alkylation of the more frequent and abundant Cys‐containing proteins. While this approach does not explicitly differentiate for oxidoreductase enzymes, it should be mentioned in this context since a large share of the 25 human selenoproteins have been assigned as oxidases or reductases.^[55]^

## 3 Conclusion and Future Perspectives

The examples highlighted in this minireview have shown that for a few of the most important classes of oxidases ABPP probes have been developed. The probes **1** for P450s, **13** for aerobic flavoproteins, and **33** for aldehyde dehydrogenases are rather general. These compounds follow the general aim in ABPP, providing a universal probe that reacts with most members of the enzyme family to study. This approach could be used for metabolic profiling and comparative ABPP addressing the target‐specificity of small‐molecule inhibitors. In contrast, it is also desirable to have highly specific probes, allowing to measure the activity of a specific protein and, in the case of a related covalent inhibitor, determine the mode of action and target profile of such a drug. The quite specific probes **9** and **12** for MAO, **20** for MPO, and **30** for retinaldehyde dehydrogenases fulfill this second goal of ABPP research.

It has to be noted that all examples highlighted in this minireview describe the ABPP of oxidases. So far, no ABPP for reductases has been reported. Mechanistically it is more straightforward to design a warhead for an activity‐based probe for an oxidase, as by oxidation of a functional group very often a more electrophilic group is formed (e. g., alcohol to aldehyde, amine to iminium ion, etc.).

As the field is still at the beginning, there are many opportunities for future research, especially the terra incognita of ABPP of reductases has to be conquered. The work of Sieber with PLP enzymes has shown that with functionalized cofactor‐mimics a global understanding of the interactome of a cofactor on proteomic scale could be achieved. This approach could be of special interest for application to oxidoreductases as many of these enzymes are cofactor‐dependent and a suitable probe could stimulate the functional annotation of so far uncharacterized proteins. It will be exciting to see how both newly developed pan‐family ABPP probes as well as protein‐specific ABPP probes will answer unsolved biological questions in the future.

**Note added in proof**

The group of Sieber very recently reported an ABPP and photoaffinity labelling study in which they could show that the MAO inhibitor tranylcypromine (analogous to compound **35** discussed above) shows promiscuos protein labelling.

## Conflict of interest

The authors declare no conflict of interest.

## Biographical Information

*Rita Fuerst studied chemistry at the University of Vienna, where she also received her PhD in the field of synthetic organic chemistry in the group of Prof. Johann Mulzer. She did her postdoctoral research with Prof. William Roush at the Scripps Research Institute, Florida. Since 2020, she has been a Senior Scientist in the group of Prof. Rolf Breinbauer at Graz University of Technology*.

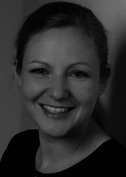

## Biographical Information

*Rolf Breinbauer studied chemistry in Vienna and Heidelberg and received his PhD at the MPI für Kohlenforschung in Mülheim/Ruhr (Germany) with M. T. Reetz. After post‐doctoral studies at Harvard University with E. N. Jacobsen, he started his independent career at the MPI of Molecular Physiology in Dortmund (Germany). Since 2007, he has been a full professor of Organic Chemistry at Graz University of Technology (Austria)*.